# Resilience and adaptive capacities of societies: social and cultural strategies

**DOI:** 10.3389/fsoc.2025.1581631

**Published:** 2025-07-23

**Authors:** Francesca Cubeddu, Elvira Martini

**Affiliations:** ^1^Institute for Research on Population and Social Policies (IRPPS), National Research Council (CNR), Rome, Italy; ^2^“G. Fortunato” University, Benevento, Italy

**Keywords:** resilience, community, strategy, innovation, networks

## Abstract

The concept of resilience is a topic that has returned to the forefront of social system analysis, especially with regard to the way in which communities react and how they can be a driving force for social reactivation as a dynamic process involving positive adaptation in the face of significant adversity. Communities have always played a fundamental role in social construction and in the implementation of actions for growth and social regulation. The same relational dynamics determined by the community involve the construction of resilience processes that guide individuals in the manifestation of their choices. The mechanisms that determine community dynamics are provided by social culture, which in turn determines both the mechanisms of resilience and those of social empowerment for community development and the possibility of creating and spreading innovation. This paper aims to analyze the concept of resilience as a social and cultural strategy of a community or group to cope with various social problems. For this reason, the text will be divided into two parts: a first theoretical part where the concept of resilience and its strategies will be defined, and a second part where, through the analysis of case studies on different social issues, we will observe how different communities have reacted and what kind of actions they have taken to restore their social functions through change and adaptation.

## 1 Resilience as an adaptive capacity of the social system

In recent years, the concept of resilience, especially associated with issues of urban and territorial systems development, as well as the dynamics triggered by the COVID-19 crisis, has been widely disseminated and is a key thought in many international and European policies. In particular, reference to the concept of eco-system resilience is made, understood as the property of complex systems to react to stress phenomena, activating response and adaptation strategies in order to restore functioning mechanisms (Martini, 2025, pp. 52–54).

Resilient systems, when faced with stress, react by renewing themselves but maintaining their functionality and recognisability (Gunderson and Holling, 1995; Gunderson and Holling, 2002). Resilience therefore does not imply the restoration of an initial state, but the restoration of functionality through change and adaptation^1^ and can be analyzed from different levels (individual, household, group, community and corporate) and from different components (built environment, economy and organizations, institutions and the natural environment), without there being an automatic correspondence between components or levels. Each level affects the components in different ways. For example, the psychological and sociological literature focusing on resilience at the community level presents qualitative distinctions between the components: (1) the equity and distribution of economic resources (Norris et al., 2008; Schlör et al., 2018); (2) the quality of the community’s network relationships (participation, connection to places, feeling of community, ability to activate resources, etc.) (Schwarz et al., 2011; Norris et al., 2008); (3) community experience, such as adaptability and creativity (political partnership, problem-solving skills, ability to act as a community, and so on (Caputo et al., 2015; Schipper, 2007); and (4) communications and information, including shared narratives, responsible media rather than reliable sources of information (Norris et al., 2008).

Other classifications assign more importance to dimensions based on the value of political-institutional and socio-health aspects or the presence of services and infrastructure. Historical consideration regarding resilience, for instance, has outlined the role of the extension of commons and their influence on social adaptation processes and culture (Van Bavel and Curtis, 2016; Rao and Greve, 2018). From the perspective of cultural anthropology, resilience has a semiotic value that is inherently conveyed by a message, description or representation. In this view, landscape is the cultural message that is perceived and communicated by local communities. The cultural and linguistic dimension of resilience presents a more complex and intricate development than engineering interpretations: it is a complex dynamic process that leads the community to discern its environment, recognizing the memory and legacy of previous generations, enriching the present. The multiplicity of interpretations has led to a flourishing use of this term for different purposes with some overlapping. In each definition, there is the metaphor of “rebound” or “return” to a new condition of “non-dynamic equilibrium.” The above conceptual clarification is necessary in order to understand the development of hybrid approaches to research that, on the one hand, fuels confusion in the interpretation of these concepts and, on the other hand, promotes the development of scientific debate on research methodologies, analysis and measurement of these phenomena (Brunetta et al., 2019).

The ecological idea of resilience dates back 50 years to an article by Holling (1973) and is a concept based on multiple equilibria. This means that ecosystems are able to respond to disturbances by changing their structure and functioning for a new system. The idea of multiple equilibria is best suited to metropolitan areas because regions have to reinvent themselves in the face of challenges. When industrial jobs disappear, regions cannot simply reinvest in manufacturing in the hope of recreating a prosperous economy based on heavy industry. Instead, they must reinvent themselves to find a new profitable niche in the global economy (Swanstrom, 2008, p. 5).

In this sense, resilience has three distinctive characteristics:

- the amount of change a system can undergo (and, therefore, the amount of stress it can sustain) and maintain the same controls on function and structure (still be in the same configuration, within the same domain of attraction);
- the degree to which the system is capable of self-organization. The resilience of a region is the ability of a community to continue its development by organizing itself against external shocks that may increase its vulnerability and even its existence. When managers control certain variables in a system, they create inter-variable feedbacks that would not be present without their intervention. The more the system is “self-organized,” the less managers have to introduce feedback. Furthermore, if the system is strongly self-organizing, those feedbacks that have to be constructed by managers are not “sensitive” or “delicate,” as there can be a significant error in the feedback induced by the manager without the system deviating from the desired behavior;
- the degree to which the system expresses the capacity to learn and adapt (Walker and Meyers, 2004).

Resilience, therefore, is the potential of a system to remain in a particular configuration and maintain its feedback and functions and involves the system’s ability to reorganize itself following changes due to perturbations. Building the resilience of a desired system configuration requires the improvement of structures and processes (social, ecological, economic) that allow for reorganization following a disruption. It also requires an important assumption of responsibility in maintaining its acquired status over time, highlighting the crucial role of adaptive capacity in ensuring the survival and success of such systems in increasingly complex and changing contexts (Martini, 2012). The adaptive capacity of systems is strictly dependent on the presence of a wide range of resources, strengthened social support networks, effective leadership, the ability to constantly learn from past and evolving experiences, and a set of well-structured institutions that adopt social foresight strategies (Arnaldi and Poli, 2012) and enable social systems to assimilate, adapt and convert quickly and efficiently as a response to the innumerable and multifaceted concomitant challenges (Martini and Vespasiano, 2015). Thus, community resilience (Marzulli and Pavesi, 2022) is something that can be acquired, provided there are actors in the community who have the will to achieve this. Resilient communities are characterized by the fact that individuals living in them perceive that social relations are strong, close and widespread; in other words, it means that people feel that they belong to their community, that they have easy access to services and programs that they may need, and that they feel they have someone (a neighbor, friend or social worker) who can help them in their time of need.

Resilient communities are also those in which service providers from different sectors (e.g., health system, education and private sector) collaborate efficiently and effectively by sharing human and material resources and avoiding duplication and fragmentation of services. Another aspect that defines a resilient and responsible community is that of respecting different linguistic, ethnic and religious backgrounds. Resilient communities are those that create programs and services and build infrastructures that promote—rather than repress—linguistic, cultural, ethnic and religious diversity. In practice, resilience in social systems can be applied through various strategies and interventions. For example, it can be implemented through community education and training (Malaguti, 2020), in order to increase awareness and preparedness to deal with crisis situations. Furthermore, the adoption and implementation of public policies aimed at promoting social inclusion and community cohesion can contribute to the resilience of social systems. Similarly, investing in disaster risk reduction infrastructure and strengthening social support networks can positively influence the adaptive and resilience of communities during stress and shock situations. Finally, collaboration between different actors, including local governments, non-governmental organizations and the private sector, can foster the creation of synergies and the sharing of resources to improve the resilience of social systems (Scantamburlo, 2024). Resilience is a concept of considerable richness that, if approached with the correct complexity, can be the key to an effective process of cultural innovation and approach to the construction of design and management processes of territorial and urban solutions. However, in the current landscape of practices one can find an application of resilience that recalls more the “engineering” dimensions, tending to develop strategies aimed at specific issues and problems (climate change, solution of specific environmental vulnerabilities) through the optimization of urban metabolisms and a greater attention to the build environment and technological innovation aspects (Colucci and Cottino, 2015, p. 37). Furthermore, there is a tendency to place too much emphasis on the adaptive capacities of communities, neglecting the structural factors and social inequalities that can influence a system’s ability to cope with crises. Some scholars also criticize the use of resilience as a tool to blame societal problems on the inability of individuals to adapt, instead of addressing the structural causes of problems. Controversy also surrounds the overemphasis on optimism and the ability to overcome difficulties, while ignoring the mental and emotional scars that can result from stressful and traumatic events. Therefore, reflection on community and system resilience should be addressed by addressing the multiple dimensions of transition and adaptation processes, the considerable vulnerabilities of urban socio-ecological systems, the relationships between social equity and environmental sustainability, and by envisaging an assessment of the possible effects (direct and indirect) that are activated in the social, governance, environmental and economic components (Colucci and Cottino, 2015, pp. 37–38).

The concept of resilience as an attribution of responsibility to the adaptive capacities of social systems is of fundamental importance in understanding how communities can face and overcome challenges (Beretta, 2022). It is evident that the resilience of social systems depends on a number of factors, including resources, cultures, social and political structures. In this sense, it is an interactive concept, which cannot be measured directly, but must be inferred from individual variations in response to significant levels of stress or adversity. It can manifest itself in various ways, such as the maintenance of stable functioning (sustainability), recovery after an initial stress response (rebound) and adaptation or transformation of existing structures (Ungar, 2019): there are, therefore, no one-dimensional answers to the questions of how individuals respond and adapt to changing conditions. For these reasons, it will be important for the future to further deepen resilience assessment methodologies and develop improved practical applications to support social systems in improving their predictive and adaptive capacity, remembering that a resilient community is first and foremost a supportive, autonomous community with a strong social cohesion, endowed with a sense of belonging and collective memory, capable of taking responsibility both individually and collectively. Which interactions are most likely to act as a catalyst for resilience will depend on the nature of the adversity encountered, the level of response acted upon, the timing of the adversity and the broader context in which these interactions take place.^2^ Furthermore, the meaning and use of the term resilience aims to deepen understanding of how the term came to be adopted in disaster risk reduction and to resolve some of the conflicts and controversies that have arisen during its use (Alexander, 2013). The aim of this work is to use case studies to show how communities respond to extreme social and natural events by activating their resilience capacity learned from a cultural process inherent in the social system. The qualitative case studies aim to highlight the effects of different disasters on the population. The expression of social resilience in communities.

The concept of resilience, now increasingly used in the debate on the innovation of intervention models for territorial development, closely concerns the territory and third sector organizations, especially those committed to working together with local communities to solve emerging problems and needs. The term resilience in fact relates to the change in approach deemed necessary to continue guaranteeing prospects of sustainability, in the face of those environmental, economic and social vulnerabilities that have rapidly invested (and continue to invest) the living context of local communities (Martini, 2025, pp. 56–58).

It is possible to define a system as inherently vulnerable to stress and shocks and minimally resilient, as defined by Folke (2006, 2016). In this sense, vulnerability is opposed to resilience, even though they act simultaneously. However, there is a significant difference between vulnerability and resilience: the former includes exposure to a specific hazard, while the latter emerges from the characteristics of a complex interaction between the system itself and the interaction between society and its governance. Resilience is more than just vulnerability,^3^ not only including the analysis of exposure to stress and shocks but also defining periods of recovery and reorganization that include community and cultural instances later considered the “assets” of urban systems. In Brunetta et al. (2019) study on the resilience of “socio-ecological and technological systems (SETSs),” we read how the interaction of social systems with their environment is the basic condition of community resilience (Folke, 2006). This interaction does not require a hierarchical relationship between the components (society, environment and infrastructure): SETSs are neither the socio-technical component of ecosystems nor the natural environment of social groups and their settlements (Gunderson, 2010). They are complex systems in which the integration between socio-technical and biophysical factors is reciprocal and flows in a continuous process of adaptive co-evolution (Redman, 2014).

The complexity of the resilience process is such that it requires a new point of view that considers the positive or negative feedback between the community (Beel et al., 2017), its heritage and relationship with the environment. Feedback is a fundamental endogenous process that differentiates community resilience and strengthens the ability of a social group to respond. From this perspective, cultural and natural heritage are fundamental to maintaining a community’s memory and sense of belonging, and therefore its maintenance or enhancement profoundly increases a community’s cohesion and resilience. In turn, cultural heritage is a resource that depends on the structural characteristics of settlements, their maintenance and state of obsolescence; thus, the structural resilience of buildings is based on the ability to respect traditional manufacturing knowledge. The ability to preserve know-how, routine maintenance and protection approaches of cultural heritage depend on territorial *governance*, which leads to the possibility of increasing the inherent resilience of a system. Resilience from a community perspective is then influenced by the difficulties people encounter during the recovery phase. Initially, resilience is characterized by the negative impact of a shock, whereas in later stages, feedback between people, institutional capacity to recover, learning capacity and the environment may favor different scenarios and the formation of “transitional communities” that may also be conflictual (Fenoglio, 2006).

In the studies on resilient communities, an essential contrast between the tradition of interventions oriented toward the so-called “clinical model” (which are based on the prevailing assumption that communities are incapable of handling a crisis without help from outside) and an opposite view, that of the “competent community” (according to which people are conceptualized as being able to catalyze the resources needed to cope with challenges) is first of all evident. The work of Prati and Pietrantoni (2009) explicitly aims to introduce a new perspective to theorize resilience as a “problem-oriented coping” process.

The approach is based on the idea that the community in its actions triggers a resilience process based on problem solving and resource maximization. A process of community planning in which a strategic development plan of a local community is built on the basis of the assumption of responsibility by each individual, and finally a process of *community development* (Marzulli and Pavesi, 2022) in which, thanks to social capital, it is possible to create spaces of sociality where the «participation of all citizens can be fostered as bearers of desires, needs capabilities, resources; create spaces for co-planning and co-management between public institutions, private actors and civil society; thus reduce the perceived distance between citizens and institutions» (Pavesi and Ferrari, 2020, p. 142).

A number of interesting insights come from studies dedicated to observing the ways in which societies organize themselves to cope with catastrophic situations, which are viewed as true and primary “learning opportunities” and as possible occasions for innovation in organizational *routines*. In this perspective, the resilience process is interpreted as a social learning space,^4^ in which pre-existing individual capabilities count, but even more so the collective competence (*community capability*), which can be developed on the basis of a cooperative approach.

Social resilience, from this point of view, identifies, rather than a solution, a working approach oriented toward effectively managing the process of “transition” from active minority intuitions that grasp elements of value from discontinuity, to actual organizational models in which the community itself is found and recognized. Social resilience (or community resilience) presupposes a strong orientation toward experimentation and a significant readiness to flexibly manage the process. Unlike intervention logics suited to known problematic situations and stereotypical interaction contexts, in which “one knows where to start from and where to go” (Cottino, 2009), in this type of circumstance the “transition” that one proposes to govern through community mobilization is in fact that between a known problematic situation, but one that is difficult to interpret, and a vision of the future that is destined to take on clearer connotations only in the course of its implementation. From this point of view, the process of community resilience binds itself less to the usual models of social planning and design and more to “entrepreneurial” logic, understood as pioneering activation to try to turn an intuition into a project. It seems, therefore, particularly appropriate in this case to speak of “social enterprise”: not only and not so much in relation to the (potential) capacity of the initiative to tackle a social problem but because of the (actual) capacity of the initiative to activate multiple components of local society. Social enterprise as collective enterprise, therefore, also and above all because it presupposes a socialization of risk before (and in function of) a socialization of benefits (Colucci and Cottino, 2015, pp. 37–38).

In conclusion, the community plays an important role because it guarantees, through its system of relationships, both an approach of solidarity during difficulties and social responsibility for social needs and well-being, for the production of a social product defined by the community and the shared culture (Berger and Lukmann, 1966). In the community there is an underlying bond of shared social welfare that must be found in times of difficulty. The community allows a condition of support and resilience in situations of risk, emergency and crisis and at the same time it allows, through dynamics of participation and collaboration, social reconstruction guaranteeing the reproducibility of the social system (Colazzo and Manfreda, 2019). Finally, the community itself ensures a culture of resilience and the tools of a social awareness, determined by the social responsibility to manage the effects of risk and emergency events (Manyena, 2006; Cubeddu and Mangone, 2024).

## 2 Chief resilience officer for implementations of resilience strategies

The importance of the role of urban communities in the contemporary context focuses on the importance of addressing the challenges and opportunities that characterize modern cities: ensuring the resilience and sustainability of urban areas, considering the social, economic and environmental changes taking place. This context requires careful planning and efficient management of resources, as well as appropriate leadership capable of guiding urban communities toward a more secure and prosperous future.

The context of modern cities is characterized by rapid urbanization processes, social and economic complexity, along with challenges such as climate change, air pollution, water pollution, and ever-increasing demographic pressures.

Cities have to face these challenges in a responsible manner, adapting and preparing for emergencies and crises efficiently and effectively. In addition, the need to preserve citizens’ quality of life and the environment calls for an innovative, more participatory and resilience-oriented approach (Poli et al., 2022; Martinelli and Mininni, 2021), which allows for the prudent management of resources, the promotion of renewable energy, and the creation of a safe, sustainable and inclusive urban habitat for all its inhabitants. This implies the planning of accessible green spaces, the promotion of sustainable mobility, the adoption of smart technologies for environmental monitoring and the management of public services, and collaboration between the various public institutions, the private sector and the local community (Vitale, 2024; Fornari, 2018).

As already noted, resilience refers to the ability of a *city/community* to withstand, adapt to and recover from shocks and stresses, such as natural disasters, economic crises or social conflicts. It implies the ability to rebuild, strengthen and improve the physical and digital infrastructure, economy and social cohesion to ensure the safety and well-being of citizens. Urban resilience is not only a matter of responding to traumatic events, but also of promoting policies, practices and processes that increase the capacity of cities to manage future challenges in a sustainable, inclusive and efficient manner (Piperata, 2024).

This requires the active participation of inhabitants, businesses and territorial institutions in the planning and implementation of resilience strategies, as well as the involvement of experts and stakeholders in the decision-making process. Furthermore, urban resilience implies a deep reflection on urban development, including the reduction of inequalities, the promotion of social and environmental justice, and the creation of safe and accessible public spaces for all. And sustainable planning is a key aspect of urban resilience, as it aims to create cities capable of sustaining long-term economic, social and environmental growth. This includes designing accessible public spaces, promoting environmentally sustainable transport, efficiently managing water and energy resources, and protecting natural habitats. Sustainable urban planning is key to ensuring that cities are able to meet future challenges in a way that is fair and sustainable for all citizens.^5^

In ensuring the realization of all this, the introduction of a recent professional figure plays a key role: the *Chief Resilience Officer* (from now on CRO) (Martini, 2025, p. 64–69). The role of the CRO has emerged as a response to the increasingly complex global challenges to which organizations are exposed. It is an evolution of traditional risk management figures, with a focus on adaptability, innovation and sustainability. The origin of this role is closely linked to the need to strategically integrate resilience within the organizational culture, ensuring its security, continuity and social responsibility. This is a very important professional figure within urban institutions, responsible for ensuring the adaptability and resilience of cities in the face of shocks and stresses. His or her key role is to effectively coordinate the various city agencies and departments, developing detailed strategies and plans to address the multiple challenges of urban resilience to ensure efficient and optimal management of available resources. The CRO carries out its work closely with a wide range of community actors, including local governments, non-governmental organizations and the private sector (promoting development models based on the Triple Helix metaphor),^6^ i.e., a high level of collaboration and exchange of knowledge and resources, in order to create a strong and cohesive network to address future challenges and promote urban resilience in a sustainable manner.

*Chief Resilience Officer*, basically responsible for identifying, developing and implementing resilience strategies within the organization. He is responsible for assessing external risks and threats, as well as creating mitigation and recovery plans. In addition, he or she oversees the coordination between different organizational functions to ensure optimal resilience management across the company.

To be effective in his or her role, the CRO must have a deep understanding of the specific needs and challenges of the urban community in which he or she operates. This includes understanding critical issues related to environmental sustainability, security, social equity and climate change, among others. A crucial task of the CRO is to facilitate and promote public participation: this implies actively involving community residents, creating platforms for dialogue, listening to their concerns and encouraging active involvement in shaping urban resilience policies and actions (De Luca, 2022). In addition, the CRO must ensure effective communication and appropriate awareness-raising on urban resilience in order to engage all sectors of the community and promote a resilient culture.

All the challenges that CROs face (climate change, economic crises, security threats, rapidly evolving technology and global interconnectedness) offer important opportunities for innovation and growth: for example, the adoption of new technologies can improve the ability to prevent and manage risks, while collaboration with other organizations and sectors can lead to new opportunities for economic and social growth. Furthermore, the ethical and social responsibility associated with this role should not be underestimated. The CRO’s ethical values and social responsibility manifest themselves first and foremost toward the community and the environment and imply the need to act in accordance with high moral principles and to consider the impact of decisions on society as a whole. This includes the promotion of diversity, fairness and transparency in business practices, and the adoption of corporate social responsibility policies.

In 2013, the Rockefeller Foundation launched the *100 Resilient Cities Challenge* program to promote cities’ concrete commitment to urban resilience and to support local governments in their efforts to adapt to the environmental, social, and economic challenges facing cities around the world.^7^ Specifically, the program aimed to support the strategic planning capacity of cities through direct funding of up to USD 1 million for the recruitment of a Chief Resilience Officer, the official called upon to assist cities in defining a strategy for urban resilience capable of acting as a link between local governments, businesses and civil society. Rome and Milan^8^ were the only two Italian cities selected in recent years by the international panel of experts to implement a resilient strategy with the support of the program. The themes of the challenge range from urban regeneration to the integration of refugees and migrants, from housing to territorial adaptation in the event of natural disasters: there are numerous topics on which each city can launch its own challenge and ask for the support of an international team of experts capable of linking practices and knowledge developed by medium and large urban contexts already involved in previous editions of the program.

In the urban context of the future, the envisaged role of the Chief Resilience Officer will be of paramount importance in ensuring the resilience of cities. It is expected that the CRO will have to take a holistic and interdisciplinary approach to managing urban resilience, coordinating actions between the various public and private entities involved. Among its main responsibilities will be to promote climate change adaptation policies, to foster the implementation of resilient infrastructure and technologies, and to facilitate the active involvement of city communities in the definition of resilience strategies. Furthermore, the CRO will need to be a catalyst for innovation and transformation, collaborating with experts and stakeholders to anticipate and address future urban challenges. It will be crucial for the CRO to develop a long-term vision for urban resilience (with typical social foresight and technological foresight approaches; Martini, 2023) and to work synergistically with different stakeholders to define sustainable solutions and risk mitigation strategies. In order to cope with a growing city population, it will be important for the CRO to focus on sustainable urban planning, water management and the adoption of policies to reduce greenhouse gas emissions. In order to ensure the effectiveness of resilience measures, it will be essential for the CRO to have a good understanding of the social and economic challenges that cities face. It will therefore be necessary to work closely with local actors to develop inclusive strategies that take into account the diverse needs and priorities of communities.

In summary, future trends in urban and organizational resilience indicate an increased focus on innovation and adaptation to global challenges such as climate change and health crises (Minet, 2020). Resilience strategies are expected to evolve, with greater integration of advanced technologies and the adoption of more flexible and adaptive approaches. Furthermore, collaboration and co-creation initiatives between organizations are expected to grow significantly in order to foster collective resilience at the community and global level. The role of the Chief Resilience Officer is thus of paramount importance in ensuring that urban communities are able to meet the challenges of the present and the future (Baglieri, 2023). The basis of his work is risk management, an element that is often still treated marginally in both the public and private spheres. Nowadays, this is a fundamental step to be integrated into administration and proof of this is the various crises faced in recent times, not least the COVID-19 emergency: the pandemic was in fact the emblem of a predictable, but unthinkable event that, if managed with the right tools and swift timing, can be contained and curbed both in terms of expansion and damage to the community. Therefore, through strategic planning, resource coordination, monitoring and evaluation, and the promotion of public participation and awareness-raising, the CRO plays a key role in guiding communities toward a more resilient future.

## 3 Toward a culture of resilience: analysis of some case studies

In the definitions of resilience, it has been observed that it is not only the response of a group or the entire community to a given event and social problem, but also the way the community itself responds according to its cultural capital, and its adaptive capacity following a given event. A community’s expression of resilience is observed in specific events, in fact, depending on the magnitude and/or the event itself, the community organizes itself or behaves differently. Many resilience actions, moreover, occur through the mutual trust (Cubeddu, 2024b) of the community, the network relationships between them and with different institutions. Trust is therefore, a promoter of social security. As resilience is not only a matter of enduring a traumatic event without damage and loss, but also the ability to learn from traumatic or stressful events, to grow, reform and regenerate through overcoming a traumatic event through experience. For this reason, the concept of resilience can be linked to the terms of social perception and security consisting of three sub-dimensions (Crowley et al., 2003): attachment, distress and control. They express the reaction of both individuals and the community at the psychological level of the effects determined by the situation of fear, security and perception.

The theoretical concepts of resilience analyzed above can be concretely observed and understood by analyzing the adaptive capacities of different communities with respect to certain events and problems of different origin: a natural event (earthquake), a social problem (educational poverty) and an environmental problem (pollution). These case studies follow a qualitative approach and seek, through precise analysis, to build conceptual validity, exploring in detail the functioning of causal mechanisms in individual cases, helping to formulate new hypotheses and taking into account complex causal relationships (George and Bennett, 2004). Bennett and Elman (2007) note that “case study methods, particularly the combination of process tracing and typological theorizing, have considerable advantages. […] The advantages of these methods lie in the study of complex, relatively unstructured and infrequent phenomena that are at the heart of the subfield” (p. 171). Case studies are fundamental to this analysis because they are an important element in identifying cultural and social modes, approaches and practices that show how communities respond and react by activating specific actions to restore their social functionality through change and adaptation. There are several case studies in the literature that highlight how different communities respond to risk and emergency events (AAVV, 2023). Different communities respond differently depending on their social and cultural context.^9^ Specifically, Italian case studies are analyzed, not only because they have been the subject of several scientific studies, but above all because Italy is one of the European countries most affected by the natural and social problems that the community has had to face. Furthermore, these cases correspond to Ronan and Johnston (2005) model of community resilience, in which a process determined by Strengthening Systems 4R (Risk Reduction, Readiness, Response, Recovery) can be observed, in which a central factor is the prevention of the consequences of disasters by strengthening the systems responsible for risk reduction, preparedness, response and recovery. In fact, there is a strong push from the population to respond actively and promptly to the situation and to be able to re-establish social order.

Analyzing the response to natural risk events (Cubeddu, 2024a), we observe how there can be two modes of attitude on the part of the population, considering an international example such as Japan, which for natural emergencies (earthquakes and tidal waves) could be considered a reference model (Cubeddu and Mangone, 2024). In fact, during the earthquake that struck the Ishikawa prefecture on 1 January 2024—with a magnitude of 7.6 –, despite the critical situation (206 victims, 100 missing and more than 50 thousand people out of their homes), the population managed to save itself by adopting the measures learned and planned, thanks also to the awareness of the trust that citizens placed in the government and in its management of the emergency (communication and training) and in future safeguard measures. In Italy, on the other hand, during the post-emergency phase of the 2009 L’Aquila earthquake—a time when the community had to “come to terms” with the effects of the earthquake—there was a central role for the community in managing resilience mechanisms. The 2009 L’Aquila earthquake still shakes the population of Abruzzo strongly; in fact, reconstruction sites and community initiatives to socialize and re-appropriate the city are still active today. With the end of the emergency phase of the L’Aquila earthquake, two phases began: the post-earthquake phase and the reconstruction phase. The former sets in motion the risk dimension of both the seismic event and a social, economic and political crisis, while the latter is still developing. A phrase that can summarize the effects of the earthquake and construct a condition of risk is the following: What used to be taken for granted is now no longer taken for granted. L’Aquila community found itself having to deal with an event for which it had not been prepared. An event that was still considered not possible and that was underestimated, both politically and scientifically.

The population has not received any form of education and communication on how to manage and cope with such events. It is true that the earthquake is not predictable and it is impossible to zero its damage and future impacts, but as observed by the Japanese case, it is possible to decrease the direct and indirect effects through a management plan. The reconstruction of L’Aquila, which is still active, fully involves the reconstruction of the L’Aquila community with its social integrity. It must be kept in mind that the community does not just lose the city with the earthquake, but its social identity. The loss of places of aggregation and socialization (squares, cultural centers, houses, etc.) means a lack of belonging to the social and cultural system and a displacement of socialization. The absence of gathering places and the growing years to rebuild the city means a loss of social integrity and cohesion. The resilience drive of the community is to build and rebuild where possible, to regain social ties and achieve social sharing of belonging. Re-appropriate memory places as much as possible to regain social consciousness. The reconstruction that is still in progress focuses on how there is a desire and need for the L’Aquila community itself to start again and rebuild, managing as soon as possible to reopen activities where it was possible and return to live in their homes or rebuild them. Returning to live in the places of memory, through the social aggregation determined by the social community. Another example of resilience in an Italian earthquake event is the 5.9 magnitude earthquake in Emilia-Romagna on 20 May 2012, where the community played a central role in earthquake risk management and especially in the reconstruction phase. Reorganizing aggregation and community cohesion. From the very first moment of the event, the people of Emilia Romagna activated the “*Do it yourself*” policy with which they facilitated and facilitated the work of Local Administrations, Civil Protection and volunteers. Emergency management actions were immediately activated, the Deputy Commissioner appointed by the Government to manage the emergency and reconstruction immediately sought to involve civil society and make it interact with the Local Administrations, so that it could take part in decisions for future reconstruction. In this earthquake, the expression of union is strength was visible and, therefore, how the will to cooperate can try to raise and revive an entire Region, considering not only the productive activities but also the morale of the citizens themselves injured by the earthquake. The sources of social aggregation of a community were rebuilt thanks to social will and also with the help of political institutions. “*We*” was the word that the Emilia-Romagna region transmitted. The battle hymn that the population chose to represent itself was Pierangelo Bertoli’s song “*A muso duro*,” which was played and sung throughout the region with the intention of communicating to all the different communities affected that “a warrior without a country and without a sword with one foot in the past and his gaze straight and open in the future.” To motivate this idea even further, a guitar was made from a wooden beam of a house that collapsed during the earthquake, representing the ability of the population of Emilia-Romagna to start again from the foundations and look to the future because it is the population itself that determines its future by going back to the way it was before (“Gazzetta dell’Emilia,” 01 November 2014—*Interview with musician Alberto Bertoli*). On a national level, in 2010, after the strong earthquake that hit L’Aquila, *Earthquake—I do not risk* initiative was launched, promoted by the Civil Protection Department and Anpas (National Association of Public Assistance), in collaboration with *Ingv* (Institute National of Geophysics and Volcanology), *ReLuis*, (Consortium of the Network of University Laboratories of Earthquake Engineering) and in agreement with the Regions and Municipalities concerned. The initiative is disseminated through the portal where information on the earthquake is provided and with a campaign in the squares of Italian cities. The aim of the campaign is to promote a culture of prevention, to train a more aware and specialized volunteer and to start a process that leads citizens to take an active role in reducing seismic risk. On set days in the squares, volunteers will be busy distributing information material and answering citizens’ questions on possible actions to be taken to reduce seismic risk.

The second event analyzed is the community’s response to a social problem such as educational poverty, which has been affecting the Italian state and the European Union in recent years. An event that can also be included among the risk dynamics. In fact, it is relevant to specify that in many areas of the world where there are still wars, political uprisings and numerous social problems, the level of educational poverty is very high (UNICEF, 2024). UNICEF (2024) observes that one in four children in the countries of the European Union is at risk of poverty or social exclusion, a situation that has worsened with the COVID-19 health emergency.

The European Union itself is trying to bring forward a social rights action plan planned for 2021, which can support countries with a high risk of poverty. Data published in 2023 by Eurostat shows that there is a growth in the levels of poverty and educational poverty in all EU countries and also that there is a problem with the lack of a social lift. The highest levels are in Bulgaria, Romania and Italy, which are also the main countries where those born into poverty remain poor. Moreover, for early school leavers, in 2023, the first country with the highest figure is Romania (16.6%), followed by Spain (13.7%), Germany (12.8%), Hungary (11.6%) and finally Italy (10.5%). It is relevant to observe—among these states—the response of Germany, which through activation both from above (the state) and from below (associations and schools), is trying to remedy the widespread problem of early school leaving. The *Child Guarantee* was introduced as a measure, which aims to improve access to social services for all children affected or at risk of social exclusion, in particular by ensuring education and assistance in school activities; implemented later with the *National Action Plan—New Opportunities for Children* in Germany. A child and youth care system proposed for an inclusive society by supporting children, young people and their families in difficult life situations. The aim is to take care not only of young people in difficulty but also of their families. In Italy, in 2023 ISTAT (2024) records 2.2 million households (8.4% of the total number of resident households) and almost 5.7 million individuals (9.7% of the total number of resident individuals) in absolute poverty. At the same time, the incidence of relative household poverty is 10.6% and more than 2.8 million households are below the threshold. Similar values in 2022 show that the condition of Italian households is stagnating in Italy. There is a slight increase in the incidence of individual relative poverty, which reaches 14.5% compared to 14.0% in 2022, a percentage value that corresponds to 8.5 million individuals. Values that also show a precarious condition for minors, who are subject to educational poverty. This refers to the inability and difficulty of a large number of young people and minors to access the right to learning, training and the development of skills, competences, the cultivation of aspirations and talents. It is not only an issue related to the violation of the right to study, but to the lack of educational opportunities, both those related to cultural enjoyment and to the right to play and sports activities. Opportunities that allow the growth of the individual, his social recognition and his belonging to the community. Educational poverty (Giancola and Salmieri, 2023) is consecutive to social and economic poverty. Living in disadvantaged social contexts, characterized by family distress, employment insecurity and material deprivation affect educational poverty as it alienates individuals from the social context they belong to and from the possibility of their expression. In this mechanism, the community tries to respond with a resilience approach to this issue. One example is the *CARE*^10^ project, which envisages a network involving teachers, families and associations. Through workshops, educational activities and support paths for families, an attempt is made to ensure inclusive and quality education for all regardless of economic and social conditions. Other examples of resilience with respect to educational poverty (Di Genova, 2023) are the educating communities both in the sense of the school community and the different actors who are committed to counteracting school drop-out through different approaches, educational methods (Chistolini, 2023) and by providing educational material to counteract school drop-out.

In addition, the *Futuro Prossimo*^11^ project—supported by the social enterprise “*Con i Bambini*,” promoted by 26 partners including local authorities, schools, third sector organizations and led by Save the Children—envisages the creation of educating communities by developing integrated inclusive educational interventions in schools, in different areas of Italy, in which through networking and educational work we try to create educational opportunities and contrast the educational and social crisis caused by poverty. The role of educating communities is the resilient response through the activation of a social network that aims not only to share cultural capital, but also to raise awareness and contrast poverty and at the same time create a culture of the future, which aims at the growth of the individual and his community.

Resilience dynamics enable communities to activate themselves and find responses that are aimed at social well-being. It is therefore relevant to observe the role of the community and that of each social actor within resilience actions (UE, 2021a,b).

In strategies and actions for resilience of change to pollution—as in the first two examples—the social actors brought into play are both the whole community but also the institutions with political actions. The role of the community is in close contact with associations and institutions.

Building resilience to environmental pollution also means fortifying an education to change one’s own actions that can affect climate, environmental and human well-being. In 1962 when Rachel Carson published the text *Silent Spring* in which for the first time the consequences of human decisions and actions on both nature and individuals were denounced. Specifically with regard to the effects of the use of DDT, on agriculture, which with toxic elements does not allow plants to be devastated by insects, and at the same time the possible toxicity to humans (Carson, 1962, pp. 172–237). Studies by Motlagh et al. (2024) show how the impacts of pollution are visible in climate change and how events are increasing in frequency and severity.

An early advertising campaign that in Carson’s book (1962) is highlighted as the “age of poisons” in which DDTs are sponsored and used by everyone precisely because there is a distorted perception of reality given by a failure to communicate the effects and the real components of the product sold. An early form of greenwashing in which an attempt was made to clean up the real negative effects. Worldwide, attention is beginning to be paid to the actions performed not only by individuals, but also by companies and businesses. Awareness is being created of the present and future repercussions on both the environment and the social system (IPCC, 2022, 2023). With respect to companies, it is the first time that the impacts produced by the products used are being observed, highlighting the importance of an awareness on the part of individuals and the state in predicting the effects and preventing the use of products that are toxic to humans and the environment. With the UN Agenda for Sustainable Development (United Nation, 2015) we see the construction of a set of different strategies for both mitigation and adaptation, aimed at affecting both the causes and consequences of climate change. Mitigation refers to all strategies proposed by plans to reduce pollutants such as greenhouse gas emissions and the encouragement of sustainable practices,^12^ the use of renewable energy sources and the promotion of energy efficiency (United Nation, 2015; ASviS, 2024). Sources of pollution are diverse and around the world there are many examples of areas heavily polluted by platelets, fossil fuels, waste, transport, pesticides, chemicals.

Globally, the Health Effects Institute (2024) records high pollution rates among the top countries in Asia. Remarkably, in a state like China, where there has been strong economic growth and is counted as a polluting state, there are examples of resilient communities where the inhabitants have tried through their actions to improve the local situation, to resist and counteract pollution. A first example is the village of Yucun where the inhabitants realized that by extracting minerals from the mine they had caused irreparable damage to their land, resulting not only in the impoverishment of green areas but also in the pollution of the water and the displacement of the population there. They decided to re-establish a balance and focus on rural development and tourism with 0 km agriculture or proximity agriculture and focus on small business.^13^

Another example, in the city of Hong Kong, is the creation by the *Clean waterwaves initiative*, an NGO set up in collaboration with the *Hsbc bank* as part of its *Clean waterways programme*, with the aim of designing a fleet of boats capable of capturing waste from the ocean, to combat marine pollution and free up the city’s beaches and harbors. Since 2022, these solar-powered boats (Solar explorer, Aqua explorer, Harbor explorer and Wayfoong explorer)—7 days a week, 24 h a day—have been sailing in Hong Kong’s harbors to clean up litter and microplastics.

In Italy, an episode of resilience actions against environmental pollution is provided by the denunciation actions carried out by the territories affected by the *ecomafia* (Legambiente, 2024a,b), all those illegal activities committed by mafia-type criminal organizations that cause damage to the environment, such as the denunciations in the Terra dei Fuochi in Campania or in Lazio in the Latina area.

In which companies pollute the surrounding area and illegally exploit land resources. Relevant in this case is the denunciation carried out by citizens and communities and the support of associations such as Legambiente and Libera, which over the years have enabled community resistance to these disasters and the re-appropriation of their territory. A different example in Italy of a return to sustainability and community resilience is the re-appropriation of green areas from situations of degradation or waste, through urban gardens. Among Italian cities, one can point to the neighborhood committees in the city of Rome,^14^ which have been engaged for several years in the creation and management of several urban gardens (around 150 located in different neighborhoods, such as Garbatella and Montagnola), so much so that the citizens themselves have drawn up regulations for their management and maintenance, asking the Rome City Council to sign up to these requests.

The international and national examples with respect to the three specific situations (a natural event, a social and environmental problem) show how resilience actions need to create a culture of resilience that is based on collaboration, networking and trust.

## 4 Conclusion

Resilience is a dynamic process (Luthar et al., 2000) and involves positive adaptation in relation to significant adversity. Resilience depends not only on the personality system of the individual but also on the cultural system (Sorokin, 1948). Resilience is a drive that comes from the community and facilitates social construction and reconstruction (Malaguti, 2005). For this reason, it is possible to speak of a culture of resilience that is proposed by the community itself and that fosters investment in programs, communication and policies, including by activating latent resources in the relationships between different actors (Rampp et al., 2019). The community is a continuous resource not only because it shows the community’s sense of belonging, its social dimension, solidarity and awareness of common interests, but it is also an element of social growth and development of social mechanisms during situations of risk, crisis and emergency. Community is based on the dynamics of relationships between social partners, institutions and individuals. The community has always played a fundamental role in the social construction and implementation of social growth and regulation.

The proposed examples of resilience show the central role of communities for its survival and social integrity. Furthermore, it is relevant to note that in order to respond to an issue, be it natural, social or environmental, two other variables are required: trust and social network. Two elements that are an integral part of the same community, that distinguish it and that underpin the same social relations. The implementation and the very success of actions and measures proposed by the community only happen through the collaboration of several social actors: communities, associations and political institutions.

Networking and trust, moreover, are two elements that highlight how community work itself is based on individual resilience and that provide the community and social level with the possibility of being able to achieve common wellbeing through shared choices and actions. Community resilience refers to communities working with local resources together with local expertise to help themselves prepare for, respond to and recover from difficult and extreme situations (Twigger-Ross et al., 2015). It is necessary for survival to understand what resources they can invest in and build their resilience for future livability. It is therefore to think of a culture of resilience in which it is possible to envisage a system prepared to resist and respond to different events (Wright, 2022). A cultural process that contemplates a relationship of different social actors, systems, and even with the integration of possible technological systems. Introduce resilience education that supports the fostering of a resilience culture and considers resilience from a collective, individual and institutional perspective. Set up training and educational pathways within institutions of all levels. Within this system is the new figure of the *Chief Resilience Officer* (CRO), a leader who develops resilience strategies, involving the different actors and planning specific actions and plans. A figure that is currently adopted by companies but that can be inserted and recognized in different areas. Talking about resilience means understanding the social and political role of different actors and at the same time the role communities play not only in resilience and immediate response but also in the strategies used for resilience, adaptation and transformation (Wright, 2022) of events into situations of social advantage. To speak of a culture of resilience means to understand about the community also, what are the elements of vulnerability and what are the capacities for emotional, social resilience and finally what are the cultural, social and economic capitals that distinguish them. So also to be able to speak of a multi-resilience (Fathi, 2022) in which there are different aspects on which the community can cope with events and through these characteristics succeed in reconstructing a social system, at the basis of which there is a community that being formed is aware of its own characteristics and of the reconstruction and regeneration plan that is to be undertaken.

## Footnotes

^1^Among the key concepts shared by many authors, some represent aspects of innovation for spatial design: creative diversity, redundancy, recognition of slow variables (and attention to the temporal variables associated with thresholds, i.e., strategies related to scenarios of restoration, adaptation and evolution), interconnections and interdependencies between the multiple levels (of complexity) of the components and hierarchical levels of complex systems, flexibility and innovation (understood as the capacity for learning, experimentation and development of rules and social design (Esposito, 2024) capable of embracing change, memory and multiple forms of knowledge.

^2^For an interesting reflection on the subject, see Schoon (2021).

^3^Many studies have focused on assessing the vulnerability and resilience of socio-ecological and technological systems (SETS) at the urban scale, including the economic assessment of multiple risks, proposing possible solutions and interventions to improve the resilience of territories. In this context, the flexibility of available solutions for a wide range of situations allows for the rigorous selection of different types of interventions to address climate change conditions (Leichenko, 2011; Ernstson et al., 2010; Tyler and Moench, 2012).

^4^It is a type of learning that is always relative and strategic, in the sense that it takes into account the starting conditions (the available resources and context data) and the type of challenge (the specific challenge) to be faced, focusing on the characteristics of the problem but also on the opportunities that may arise to reinvent the environment by operating in the spaces left vacant by the compromised status quo.

^5^*Best practices* and *case studies* concerning resilient interventions in cities offer important learning and inspiration for the implementation of urban resilience. These successful examples may include resilient infrastructure projects, sustainable urban planning, implementation of innovative technologies, community engagement and climate change adaptation initiatives. Interventions that have proven to be successful in specific urban contexts provide valuable lessons that can be applied in other cities, helping to foster a culture of resilience and improve the capacity of cities to face future challenges.

^6^For an in-depth look at how the *Triple Helix Model* works, see, among others, Martini (2011).

^7^For research, please see the link: https://resilientcitiesnetwork.org/ (last consultation 01/06/2025).

^8^Rome citizens were actively involved in defining priorities for action to be included in the resilient city plan, focused on combating air and environmental pollution, soil erosion, sea level rise and flooding. Milan, on the other hand, proposed to develop a set of actions to improve the local government’s ability to respond to emergencies related to climate change, from summer heat waves to floods. In addition to these issues, the city is committed to tackling social emergencies that have long plagued the city, such as the problem of *social housing* or fair and guaranteed access to public services, which can cause social tensions especially in the most vulnerable groups.

^9^The work we refer to is Vol. 15, No. 1 of 2023 of the journal *Jàmbá - Journal of Disaster Risk Studies* - https://jamba.org.za/index.php/jamba/issue/view/28 (last accessed on 17/06/2025).

^10^CARE Pathways, for project information see https://percorsiconibambini.it/care-verona/ (Last consultation date 30/12/2024).

^11^For information on the project, participating schools and modalities please visit https://www.savethechildren.it/cosa-facciamo/progetti/futuro-prossimo (Last consultation date 30/12/2024).

^12^Also consider the *Global Climate action* plans available at the link https://climate.ec.europa.eu/eu-action/international-action-climate-change/global-climate-action_en#documentation (data ultima consultazione il 30/01/2025) e il *Green Deal europeo* disponibile al link https://www.europarl.europa.eu/topics/it/article/20200618STO81513/green-deal-europeo-la-chiave-per-un-ue-sostenibile-e-climaticamente-neutrale (Last consultation date 30/01/2025).

^13^Documented by China Global Television Network (CGTN) at the link https://www.youtube.com/watch?v=-obpQX0PvRY (Last consultation date 30/01/2025).

^14^Also consider the site Comune di Roma: https://www.comune.roma.it/web/it/scheda-servizi.page?contentId=INF60787 (Last consultation date 16/06/2025) and the article “Urban gardens, the municipality on the hunt for new land to allocate. Call for tenders by 2025” in RomeToday: https://www.romatoday.it/politica/orti-urbani-attesa-bando-2025.html (Last consultation date 16/06/2025).
